# Visualizing the *In Vivo* Dynamics of Anti-*Leishmania* Immunity: Discoveries and Challenges

**DOI:** 10.3389/fimmu.2021.671582

**Published:** 2021-05-19

**Authors:** Romaniya Zayats, Jude E. Uzonna, Thomas T. Murooka

**Affiliations:** ^1^ Rady Faculty of Health Sciences, Department of Immunology, University of Manitoba, Winnipeg, MB, Canada; ^2^ Rady Faculty of Health Sciences, Department of Medical Microbiology and Infectious Diseases, University of Manitoba, Winnipeg, MB, Canada

**Keywords:** two-photon intravital microscopy, *Leishmania* infection, T cells, ear skin imaging, liver imaging, fluorescent reporters, macrophages

## Abstract

Intravital microscopy, such as 2-photon microscopy, is now a mainstay in immunological research to visually characterize immune cell dynamics during homeostasis and pathogen infections. This approach has been especially beneficial in describing the complex process of host immune responses to parasitic infections *in vivo*, such as *Leishmania.* Human-parasite co-evolution has endowed parasites with multiple strategies to subvert host immunity in order to establish chronic infections and ensure human-to-human transmission. While much focus has been placed on viral and bacterial infections, intravital microscopy studies during parasitic infections have been comparatively sparse. In this review, we will discuss how *in vivo* microscopy has provided important insights into the generation of innate and adaptive immunity in various organs during parasitic infections, with a primary focus on *Leishmania*. We highlight how microscopy-based approaches may be key to providing mechanistic insights into *Leishmania* persistence *in vivo* and to devise strategies for better parasite control.

## Introduction

The immune system must rapidly mobilize immune cells to appropriate tissue sites to eliminate infections, but at the same time, must be tightly regulated to prevent excessive tissue damage. This is largely achieved by the exquisite ability of immune cells to continuously survey most organs in the body, and by coordinating cell-cell communication among different cell types to generate antigen and site-specific immunity. Immune surveillance *in vivo* is orchestrated by a number of extrinsic factors that are impossible to fully recapitulate in their full complexity outside of the living organism. To address this, time-lapse intravital microscopy (IVM) has been used to capture cell motility and cell-cell interaction dynamics within physiological tissue environments, where stromal cell networks, physiological blood flow, lymphatic drainage and innervation remain intact. More recently, two-photon microscopy (2P-IVM) has emerged as the gold standard for *in vivo* imaging and represents an important imaging platform to refine and extend observations derived from cell culture studies. While initial studies focused on cellular dynamics at steady-state or using model antigen systems, more recent 2P-IVM studies have focused on visualizing immunity generation in response to natural infections, solid tumors and evaluating candidate vaccine efficacy *in vivo*.

## Two-Photon Intravital Microscopy (2P-IVM): The Gold Standard for *In Vivo* Imaging

Much of our understanding on immunity generation against pathogens have been derived from *in vitro* imaging studies and static histological analyses that fail to fully capture the biomechanical, physicochemical and immunological aspects of complex tissue environments. Two-photon microscopy is a powerful tool that allows researchers to visually characterize cellular dynamics that dictate the specificity, breadth, and magnitude of immune responses. Pioneering studies using 2P-IVM in intact (1) or explanted lymph nodes (2, 3) showed that naïve T cells displayed robust migration in the T cell zone at speeds higher than those previously reported from *in vitro* studies (3). Upon contact with antigen presenting cells (APCs) presenting cognate antigen, short-lived interactions were replaced by durable APC:T cell conjugates that lasted for up to 36-48 hours, and these prolonged contacts were crucial for proliferation, differentiation and generation of memory T cell responses (1, 2). Similar interaction dynamics were reported between thymocytes and stromal cells during thymic selection, where varying environmental cues dictated thymocyte motility behaviors, differentiation and function (4). 2P-IVM has also aided in uncovering the B cell dynamics in the germinal centers, where they were observed to be highly motile around the DC network (5), increasing their chances of encountering specific antigen and receiving necessary survival signals. Interestingly, B cells appear to compete with each other for both the antigen and T cell help (5, 6). More recent studies have incorporated fluorescent reporters to measure signal transduction in living cells, integrating changes in cell migration behaviors with signaling status (7, 8). These transformative studies have laid the foundation for *in vivo* characterizations of immune responses to a wide range of natural infections, where the nature of the pathogen dictates how adaptive immunity is generated (9–13).

**Figure 1:**
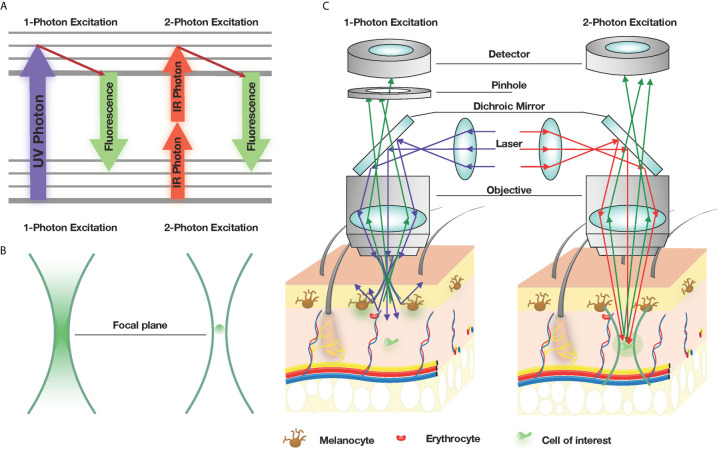
2P-microscopy is the gold standard for intravital imaging. **(A)** Jablonski diagram, illustrating the principles of excitation with one Ultraviolet (UV) photon and two Infrared (IR) photons. **(B)** Spatial confinement of signal generation. 1-photon excitation generates visible (green) signal in the entire cone of fluorescence, while 2-photon excitation generates a signal only at the focal spot. **(C)** 1-photon excitation microscopy of the skin leads to a low light penetration, which becomes scattered by melanocytes and erythrocytes, while 2-photon excitation leads to a deeper penetration will less light scattering.

The concept of exciting a molecule from the ground state to a higher energy state with two photons with identical frequencies was predicted by Marie Göppert in the 1930s (12). It wasn’t until 1990 when Wilhelm Denk developed the first two-photon microscope by combining a point-scanning microscope with an infrared mode-locked laser (14). In contrast to deactivation and emission of a fluorophore following single photon excitation, the near simultaneous absorption of two photons of higher wavelength, or half the energy, only occurs at the focal plane (14) (**Figure 1A**). This negates the need of a pinhole in front of the detection unit, as only the desired fluorophore is excited at the focal plane (**Figure 1B**). This results in significantly reduced photobleaching and phototoxicity of out-of-focus specimens and allows for 3D imaging for longer durations compared to conventional single-photon lasers (**Figure 1C**). Additionally, long wavelength near-infrared light permits deeper penetration into tissues with minimal light scattering and lower bystander absorption from water and pigment-containing proteins, such as melanocytes and heme molecules in erythrocytes (15, 16). When combined with fluorescent proteins/dyes that emit across the entire visible spectrum, availability of various fluorescent reporter mouse models and improvements in 4D analysis software capabilities, 2P-IVM approaches are becoming increasingly accessible to more research labs and applicable for a wide range of research areas.

Over the last two decades, 2P-IVM studies have helped characterize the spatiotemporal dynamics of immune responses to viral, bacterial and parasitic infections, and uncovered unique *in vivo* behaviors such as pathogen dissemination by phagocytes (17, 18), swarming responses by neutrophils to injurious insults (19–21), and bacterial reservoirs following infection (22). More recently, immune homeostasis functions of tissue-resident macrophages have been brought to light, such as their ability to rapidly sequester dead cells in the liver (23) and phagocytose inhaled bacteria in the lungs (24) in order to shield them from neutrophils and prevent further recruitment and tissue inflammation. While much focus has been placed on viral and bacterial infections, 2P-IVM studies on parasitic infections *in vivo* have been comparatively sparse. In this review, we will discuss how the utility of 2P-IVM approaches, and how this imaging modality has advanced our understanding of anti-parasitic responses *in vivo*, with a focus on adaptive immunity against the parasite *Leishmania.*

## Visualizing Anti-Parasitic Immunity Generation *In Vivo*

Despite numerous animal models that are available to study the pathophysiology of parasitic infections, this field remains vastly understudied *in vivo* compared to viral and bacterial infections. The threat of eukaryotic parasites continually expands into non-endemic countries with the rising climate temperatures, and diseases caused by these pathogens are a great threat to both human and other mammalian hosts, many of which have no cures or vaccines available. Vaccine development against parasitic infections have been hampered due to the complexity of the parasitic life cycle. For example, *Plasmodium* spp. undergo sexual and asexual stages and alternate between the human and mosquito hosts. While the live attenuated vaccine did provide protection against wild-type *Plasmodium falciparum* challenge, RTS,S, a recombinant protein vaccine, which is the only FDA-approved vaccine against a human parasitic disease of any kind to date, only has 26-50% efficacy and only provides short-term protection (25). Parasites have and continue to evolve with their hosts. Their sustained success is due to their remarkable ability to avoid detection and clearance by the host immune system. Antigenic variation, which includes continuous recombination with silent genes and epigenetic switching of sub-telomeric genes is one of the common immune evasion strategies (26). Another strategy is to encode a large number of genes that actively promote immune evasion. *Plasmodium* relies on immune dysregulation to avoid clearance, such as activation of B cells independently of T cell help by crosslinking B cell receptors with repetitive epitopes present on the circumsporozoite protein; immunosuppression through engagement of CD36 and CD51 on DCs in order to impair maturation and subsequent T cell priming; and activating immune checkpoint molecules on CD4 and CD8 T cells (27). These immunosuppressive mechanisms prevent establishment of long-term memory responses and help promote a state of persistent infection.

Leishmaniasis, a disease caused by the *Leishmania* spp., varies in severity depending on the species of *Leishmania* and the type of immune response mounted by the infected individual. Based on the parasite species, organs infected, and symptoms produced, leishmaniasis presents in three different forms: mucocutaneous, cutaneous (CL), and visceral (VL), also known as kala-azar (28) (**Table 1**). The severity of disease progression is dictated by a number of factors, including the infecting parasite dose (29, 30), composition of sand fly saliva (31), site of infection (32), degree of tissue damage (17), host skin microbiome (33, 34) and the sand fly gut microbiota (35–37). The most important parameter that dictates disease severity is the type of immune response developed by the host. Because *Leishmania* are obligate intracellular parasites and not neutralized by antibodies, patients with a predominantly humoral response are unable to control parasite load and exhibit a severe form of the disease, called diffuse cutaneous leishmaniasis (38, 39). Individuals who develop a strong CD4^+^ T cell response, characterized by high IFN*γ* production and strong delayed-type hypersensitivity reactions, are able to better control the infection, where intracellular parasites are cleared by nitric oxide produced by IFN*γ*-activated (40–42) cells of the mononuclear phagocyte system (42–46). However, an exaggerated T cell response can also lead to immunopathology which, in severe cases, can lead to mucosal leishmaniasis (47). Patients that heal their primary infections develop immunity against secondary infections but this requires the presence of a small number of persistent parasites in the lesion that remain there indefinitely (38, 48–53). Persistent parasites have been detected in three-year old skin lesions (54) as well as in extralesional sites (55, 56), and can contribute to a late cutaneous manifestation of visceral leishmaniasis, termed post-kala-azar dermal leishmaniasis (PKDL) (57, 58) in humans. Numerous factors allowing the parasites to persist have been speculated (59), and these observations indicate that while the presence of live parasites may generate immunity against re-infections, there is inherent risk for disease reactivation, especially in immunocompromised individuals (60, 61). This has been demonstrated in mice, where the expansion of Treg cells *in vivo* causes reactivation of disease in tissues at the site of primary skin infection long after it has healed (62), while depleting Tregs during the secondary challenge prevents disease reactivation at the site of infection and enhances early parasite clearance (63, 64). Thus, some of the greatest challenges in developing a vaccine against a vector-borne infection, such as *Leishmania* spp., include an incomplete understanding of complex interplay between the parasite, the host immune response and composition of the host/vector microbiome that coordinate the generation of anti-parasitic immunity.

**Table 1:** Human manifestation of leishmaniasis caused by *Leishmania* spp.

Geographical location	Subgenus	Species	Manifestation	Clinical signs and symptoms
Old world Eastern Hemisphere: Asia, Africa, and South Europe	*L. (Leishmania)*	*L. major *	CL, ML (rare)	Painless, often severely inflamed, ulcerated lesions. Heal within 2-8 months. Often present with multiple lesions which could become secondarily infected. These lesions are slow to heal and may leave disfiguring scars.
		*L. tropica *	CL, ML (rare), VL (rare)	Painless multiple dry ulcers in the skin. Heal spontaneously within a year, often leaving disfiguring scars. May lead to Leishmaniasis recidivans, a chronic form of cutaneous leishmaniasis, presenting with slowly progressing lesions which, if left untreated, become destructive and disfiguring.
		*L. aethiopica*	CL, DCL	Cutaneous nodular lesions, occasionally oronasal lesions, which distort the nostrils and lips. Progress slowly and may spread locally, taking 2-5 years to heal. Late or absent ulceration. Parasites can cause diffuse cutaneous leishmaniasis, characterized by widely disseminated nodules, most commonly on the limbs and skin. Leads to thickening of the eyebrows and resembles lupus. Does not heal and relapses frequently.
		*L. infantum, L. chagasi*	CL, ML (rare), VL (children), PKLD	Single nodular lesions with little inflammation, ulcers. Lesions heal spontaneously in a year. Splenomegaly +/- hepatomegaly, pallor of mucosal membranes. Signs of malnutrition as disease progresses. Rare complications include severe acute haemolytic anaemia, mucosal hemorrhage, and acute renal damage.
		*L. donovani*	CL, VL, PKLD, ML (rare)	Splenomegaly +/- hepatomegaly, pallor of mucosal membranes. Signs of malnutrition as disease progresses. Rare complications include severe acute haemolytic anaemia, mucosal hemorrhage, and acute renal damage. Post-kala-azar presents with hypopigmented or erythematous macules anywhere on the body, which become popular or nodular. Buccal and genital mucosa, and the conjunctiva may become affected.
New world Western Hemisphere: From Southcentral Texas to South America, excluding Chile and Uruguay.	*L. (Leishmania)*	*L. infantum*	CL, ML (rare), VL (children), PKLD (rare)	Clinically similar to old world *L. infantum* infection. Most cases occur in children under ten years of age. Some patients develop clinical visceral leishmaniasis. CL in the New World is often atypical.
		*L. mexicana, L. amazonensis*	CL, DCL (rare)	Cutaneous lesion occurring anywhere on the body post bite, which ulcerates and expands and heals spontaneously within 34 months. Diffuse cutaneous leishmaniasis is similar to Old World manifestations and does not heal spontaneously.
	*L. (Viannia)*	*L. braziliensis, L. guyanensis, L. panamensis*	CL, DCL, ML	Cutaneous lesions occurring anywhere on the body post bite, which ulcerates and expands. May involve the lymphatic system, leading to lymphadenopathy. May heal on its own after 6 months.
				Disseminated cutaneous leishmaniasis may occur, with over 20 and up to hundreds nodular or ulcerated lesions occurring without the involvement of the mucosa. Mucocutaneous manifestation can present from several months to 20 or more years after a cutaneous lesion. Nasal lesions are always present, leading to obstruction of the nostril, perforation of the septum, and eventual collapse. One third of the patients have the pharynx, palate, larynx, trachea and upper lip affected. ML almost never heals spontaneously. Secondary bacterial infections are common.
		*L. peruviana*	CL	Lesion occurring anywhere on the body post bite, which ulcerates and expands. May involve the lymphatic system, leading to lymphadenopathy. May heal on its own after 6 months.

CL, Cutaneous Leishmaniasis; ML, Mucocutaneous Leishmaniasis; VL, Visceral Leishmaniasis; DCL, Disseminated Cutaneous Leishmaniasis; PKLD, Post-Kala-Azar Dermal Leishmaniasis.

*Leishmania* parasites undergo a digenetic life-cycle, alternating between residing in a phlebotomine sand fly vector and a mammalian host (65). During an acquisition of a blood meal, an infected sand fly probes the skin and induces bleeding, regurgitating the infective metacyclic promastigote form of the parasite into the skin of the mammalian hosts, such as rodents, dogs, and humans (66, 67). The flagellated motile promastigotes are then subsequently engulfed by phagocytic cells in the skin. Neutrophils are the first cells to respond to the infection, but inflammatory monocytes and tissue macrophages are the preferred host populations, as described in detail below. Following phagocytosis, it is within the phagolysosome where the parasites transform into the non-flagellated amastigote (38). Once the sand flies ingest infected cells while acquiring another blood meal, the life cycle of the parasite completes inside the gut of the fly.

## Visualizing *Leishmania* Infection of the Liver

**Figure 2:**
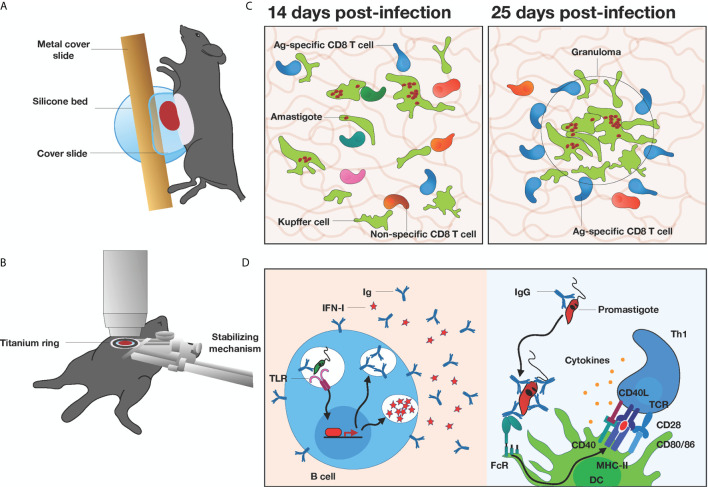
*In vivo* microscopy of the liver in *Leishmania*-infected mice **(A)** Graphical illustration of a mouse preparation for short-term liver imaging. Liver is externalized, placed onto the silicone bed, and covered with a custom metal cover slide. **(B)** Graphical illustration of a mouse preparation for long-term liver imaging. A titanium ring is surgically inserted into the mouse abdomen and connected to a custom stabilizing mechanism. **(C)** Granuloma formation in the liver at 14 vs 25 days post infection with *Leishmania donovani*. CD8^+^ T cells are recruited to the granuloma irrespective of antigen specificity, but antigen specific CD8^+^ T cells are retained at 25 days. **(D)** Pathogenic and protective roles of B cells in *Leishmania* immunity. *Leishmania donovani* can trigger endosomal TLR stimulation, induce hypergammaglobulinemia and increase type I interferons (IFN-I) expression (left panel). IgGs from B cells facilitate opsonization of *Leishmania major* parasites by DCs *via* Fc receptors to drive effector Th1 activation (right panel).

In order to complete their lifecycle, *Leishmania* parasites replicate rapidly in the mammalian host to facilitate transmission back into the insect vector. *L. donovani, L. infantum, and L. chagasi*, which all cause visceral leishmaniasis, can disseminate into the liver, spleen, and bone marrow and cause significant pathology in these organs. The liver is accessible by IVM, as extensive surgery is not required to expose the organ for imaging. For most short-term imaging studies, the liver of anesthetized mice is accessed through a small incision in the abdomen, turning the mouse on its side, gently letting the organ roll out with cotton swabs onto a silicone bed, and placing a custom metal cover slide on top (68) (**Figure 2A**). For long-term studies, an abdominal viewing window made from a titanium ring and a coverslip (69) is surgically implanted into the mouse abdomen (**Figure 2B**). In both cases, it is imperative to avoid inducing tissue damage while stabilizing the liver to remove breathing artifacts during intravital imaging.

Granuloma formation is the hallmark of leishmaniasis response in the liver and it consists of mononuclear cells surrounding the infection sites and limiting the spread of parasites. It has been long speculated that liver granulomas form around Kupffer cells, the dominant phagocytic population in the liver, but extensive *in vitro* studies were not able to confirm their role in antigen presentation within granulomas. Using 2PM-IVM, Beattie et al. showed that Kupffer cells infected with *L. donovani in vivo* engaged in long-lasting interactions with antigen-specific CD8^+^ T cells, and they were a key player in driving hepatic immunity to infection (70). They visualized, for the first time, motile Kupffer cells migrating from the sinusoids to form almost the entire core of the infected granuloma. They also showed Kupffer cell participation in antigen-specific T cell activation, localized only to the granuloma site itself (**Figure 2C**). Using hCD2.GFP reporter mice, they visualized the entire T cell and NK cell repertoire of the granuloma and found T cells accumulating around the Kupffer cells from 14 days post-infection. Together they formed granulomas which were smaller and more frequent during the early time points, and larger at 25 days post-infection. The exit rate of cells from the granuloma was influenced by the presence or absence of cognate antigen, but not the entrance rate, indicating antigen-specific retention of the cells since antigen-specific T cells migrated at lower velocities. These studies provided important insights into the dynamics of liver granuloma function, and the behavior of CD8^+^ T cells responses within these infection sites. Additionally, the same group characterized the dynamic behaviors of infected vs non-infected Kupffer using TdTomato *L. donovani* and 2P-IVM to visually identify active infection of the cells (71). Interestingly, inoculation with live parasite amastigotes led to a significant decrease in membrane fluctuation in both infected and uninfected cells, indicating that these changes are not a direct response to intracellular infection itself, but rather due to signals derived from inflammation and bystander activation. These findings corroborate the importance of the local inflammatory milieu that are not adequately recapitulated in *in vitro* studies of host-pathogen interactions.

B cell responses are considered to play a minor role in anti-*Leishmania* immunity and have been mostly studied in the context of cutaneous leishmaniasis *in vivo* (72, 73). However, in visceral leishmaniasis, Moore et al. demonstrated that highly motile B cells are present in *L. donovani* granulomas in the liver, irrespective of their antigen specificity or their capacity to interact with the intra-granuloma T cells (74). However, B cells from naïve and infected mice displayed similar levels of CCR6 in granulomas within 12 hours of adoptive transfer, suggesting that B cells passively enter the granulomas. It is unclear whether B cells are retained within the granulomas or eventually re-enter the circulation, but using an approach to measure red blood cell velocity in hepatic vessels may help address this question (68). A more recent study demonstrated that *L. donovani* can directly activate B cells *via* endosomal TLR stimulation, proposing a novel parasite-driven survival mechanism by inducing hypergammaglobulinemia and increased levels of type I interferons, both associated with immune suppression and disease pathology (75) (**Figure 2D**). This study highlights the potential role B cells play in exacerbating disease, but this is still controversial, as both pathogenic and protective roles of B cells to other forms of *Leishmaniasis* have been described (72). Antibodies and serum are not protective against leishmaniasis (73, 76), and when donor CD4^+^ T cells are transferred into irradiated Balb/c mice lacking B cells, they become resistant to *Leishmania tropica* infection (77). However, IgG-mediated opsonization of *L. major* was shown to promote antigen uptake by DCs, resulting in an increased Th1 response (78) (**Figure 2D**). Collectively, while B cells are present at the site of *Leishmania* infection in the liver, their exact role in anti-parasitic immunity or disease progression remains elusive.

While *Leishmania donovani* infection is generally associated with severe liver and spleen pathology, live parasites have also been detected in the brain (79). Here, using a combination of bioluminescent *L. donovani* and RT-qPCR, Melo et al. detected live parasites in the brain as early as three days post-infection, accompanied by a dual-phase inflammatory response (79). The use of 3D micro-CT was able to pinpoint parasites in the cranial cavity during this early phase of inflammation, which was characterized by CXCL10/CXCR3 and CCL7/CCR2 upregulation and recruitment of neutrophils and Ly6C^high^ monocytes. Collectively, these studies provide evidence that *L. donovani* can infect and inflame the brain and highlight the utility of 2D-3D bioluminescence approaches to detect parasite infections at peripheral sites *in vivo*.

## Visualizing immunity to cutaneous Leishmaniasis in the skin during acute responses

### Cellular dynamics of innate immunity generation to Leishmania infection

**Figure 3:**
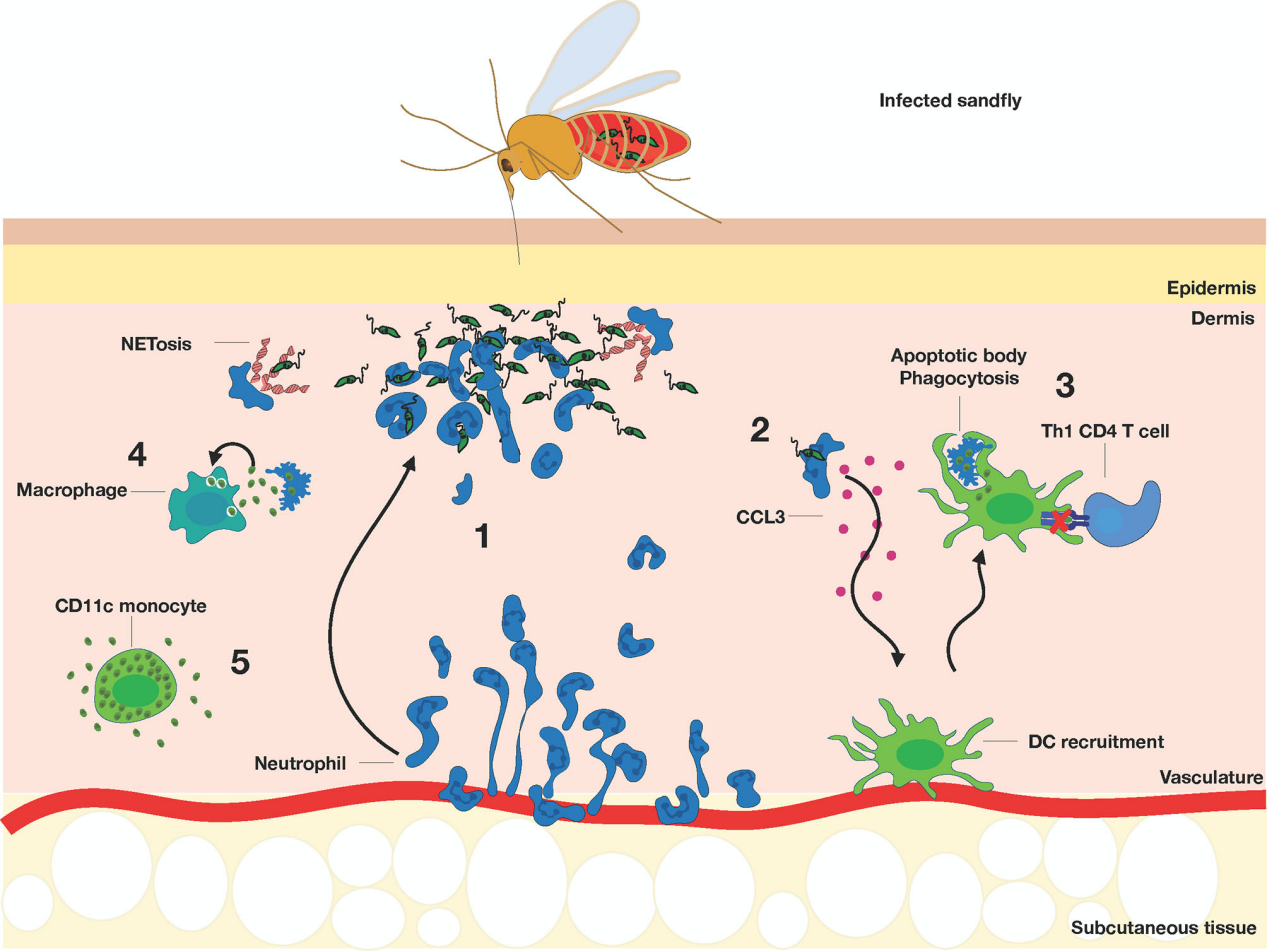
Acute response to *Leishmania* infection by a sandfly bite. (1) Neutrophils (dark blue) are recruited from the blood to the site of infection, undergo NETosis, and phagocytose the promastigotes. (2) Infected neutrophils recruit DCs (green) by producing CCL3, which subsequently engulf the apoptotic bodies of infected neutrophils and (3) lose their ability to effectively activate Th1 response (light blue). (4) Macrophages (dark green) become infected by the parasites released by the dying neutrophils. (5) CD11c^+^ monocytes (green) are highly permissive to parasite replication and further promote infection.

The ability of *Leishmania* to access host phagocytic cells to establish a chronic infection is central to their intracellular lifestyle. The first *in vivo* characterization of the sequence of events after *Leishmania major* infection was demonstrated by Peters et al., where parasites were rapidly engulfed by swarming neutrophils moments after bites from an infected sand fly in the ear (17) (**Figure 3**). As neutrophil recruitment and subsequent plug formation was observed in the absence of parasites, this initial response was speculated to occur as a result of the skin piercing damage and not the presence of the parasites themselves (17, 80), although TLR2 triggering on non-hematopoietic cells has also been shown to increase recruitment (81). Phagocytosed parasites survive within neutrophils by modulating their ability to kill intracellular parasites, such as hinder their ability to produce reactive oxygen species (ROS) and thus represents a safe haven niche for *L. major* replication (82). It is important to note that neutrophils can have a protective role, depending on the infection. Neutrophils are protective against *L. amazonensis* infection which are highly susceptible to histone-dependent killing (83–85). *L. braziliensis* parasite load was increased after neutrophil depletion in Balb/c mice (86) and amastigotes are killed by neutrophils *in vitro* (87), indicating that neutrophils are protective in this infection.

Initial studies showed that *L. major-*infected neutrophils increase CCL3 production (88) which help guide dermal DCs to the site of infection to aid with clearance of infected apoptotic bodies (89). 2PTM studies revealed that CD11c-YFP^+^ dermal DCs transitioned from an actively crawling phenotype at steady-state to arrest during *L. major* infection, accompanied by rapid capture of parasites using long, motile pseudopods (90). More recent studies have collectively shown that monocyte-derived phagocytes are rapidly recruited and make up the majority of *Leishmania* infected cells within the first few days (36, 43, 45, 91). A large influx of inflammatory CD11b^+^Ly6C^hi^CX_3_CR1^+^ monocytes into the primary infection site are observed and make up a large fraction of infected cells by day 2 post-infection, whereas these populations mediate efficient containment during secondary infections (45). Similarly, monocyte-derived dendritic cell differentiate in the infected skin upon recruitment and drive the generation of protective Th1 immunity against *L. major* (44). Interestingly, Hurrell et al. showed that infection with *Leishmania mexicana* induced rapid neutrophil influx and NET release that sequestered but did not impair parasite survival. Instead, the presence of neutrophils impaired early recruitment of monocytes and dendritic cells, delaying adaptive immunity generation through reduced CCL2, CCL3 and CCL5 expression (92). Poor recruitment of monocytes and DCs *in vivo* (93, 94), coupled with low expansion of responding Th1 cells is associated with progressive, non-healing lesion (95–98). These studies support the idea that *Leishmania* can manipulate host innate responses to facilitate recruitment of susceptible cells, even though some of these cells have leishmanicidal activity (45). Taking advantage of the fact that *L. major* parasites divide once every 15-60 hours inside host cells (99–101), Heyde et al. used a pathogen-encoded biosensor to describe a monocyte-derived dendritic cell-like phagocyte subset which were highly permissive to *L. major* replication during acute infection (102). This biosensor system is based on the photoconvertible protein mKikume, which displays green fluorescence in their native form and photoconvert to red fluorescence after excitation with 405 nm light (103). Because recently divided parasites are green, this allowed researchers to monitor parasite proliferation rates by calculating the green/red color ratio. Intravital microscopy of the ears at three weeks post-infection revealed clusters of highly proliferating parasites that were distinct from clusters of low-proliferating parasites, indicating that different cellular niches dictated parasite proliferation and persistence. Imaging studies revealed that parasite proliferation rates vary depending on the phagocyte host, where Ly6C^+^CCR2^+^ CD11c-expressing monocytes were the main reservoir for the most rapidly-proliferating parasites during acute infection (102). While viable *L. major* parasites can be seen released by apoptotic neutrophils in the vicinity of surrounding macrophages (17), direct cell-to-cell transmission was also captured by IVM imaging. Using the non-healing *L. major* Ryan strain, infected neutrophils can transmit infection to dermal tissue resident macrophages *via* efferocytosis, or phagocytosis of apoptotic cells, in the first 24 hours of infection (104). In these studies, dermal tissue resident macrophages were initially infected after sand fly transmitted infection, while infection transitioned to myeloid cells including inflammatory monocytes and monocyte-derived dendritic cells by day 12 post-infection. While differences in the parasite dissemination kinetics and target cells involved can be attributed to differences in the *Leishmania* strain used, infection route and parasite dose, these studies collectively illustrate the various strategies *Leishmania* employs to hijack physiological phagocytic responses and cell-cell communicative behaviors to access, replicate and disseminate infection to various phagocytic cell subsets *in vivo*.

**Figure 4:**
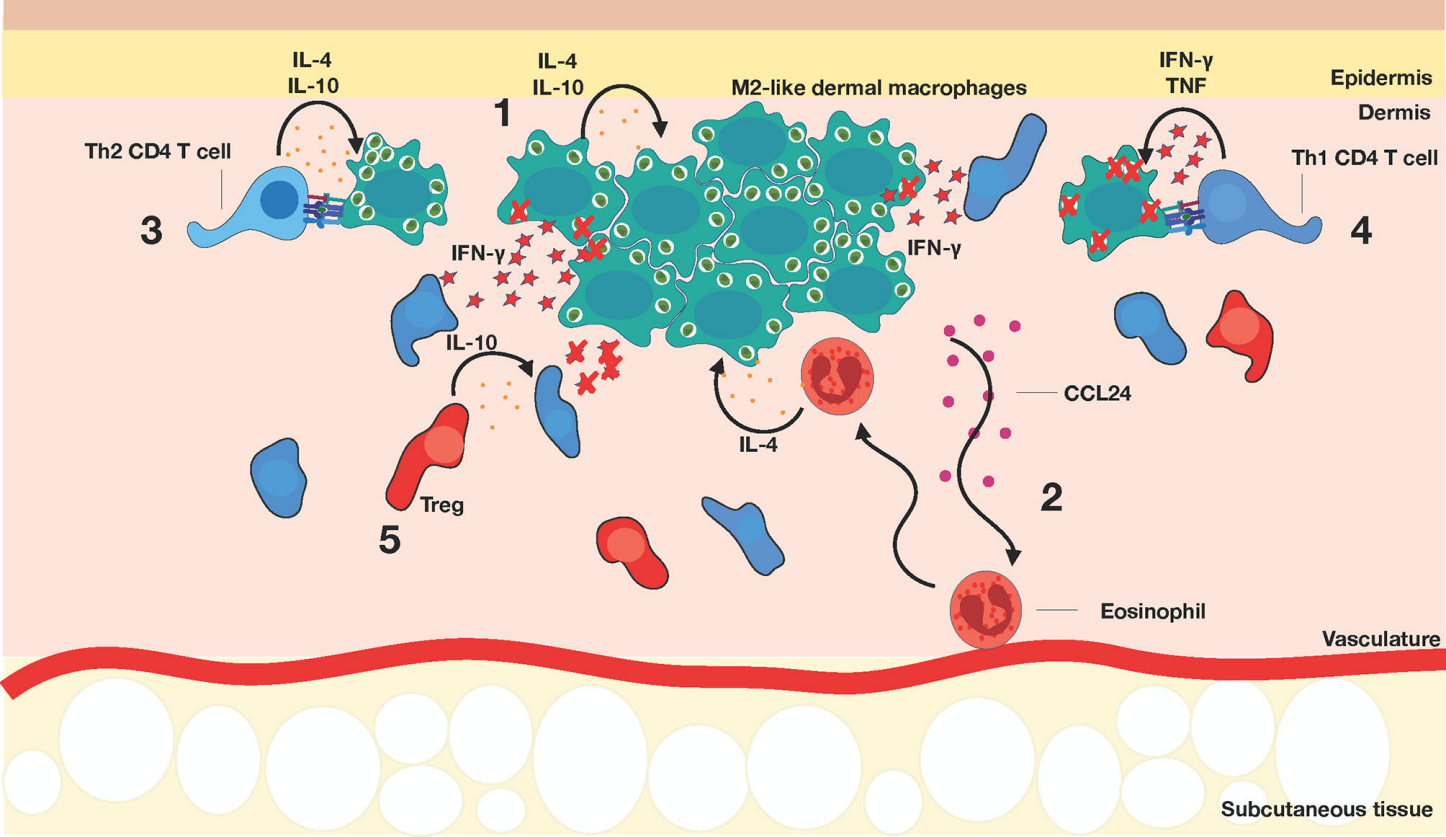
Establishment of chronic *Leishmania* infection. (1) M2-like dermal macrophages (dark green) harbor parasites. (2) Eosinophils (red) are recruited to the site of infection *via* CCL24 and produce IL-4 to maintain the M2-like dermal macrophages. (3) Th2 CD4^+^ T cells (light blue) exacerbate disease progression, while (4) Th1 CD4^+^ T cells (dark blue) stimulate macrophages to improve leishmanicidal activity, mediated primarily by IFN*γ* production. At this stage of infection, Th1 CD4^+^ T cells tend to surround but do not enter the lesion. (5) Regulatory T cells (red) are recruited to the lesion site and can suppress Th1 responses, possibly through IL-10 production.

*Leishmania* can circumvent anti-parasitic strategies of phagocytic cells, thereby creating a suitable cellular niche for long-term survival that is shielded from the immune response. *Leishmania* survives within phagolysosomal vesicles and depend on Th1 cells for cellular activation and parasite clearance (38, 105, 106) (**Figure 4**). Studies have shown that transition of intracellular parasites from the promastigote to the amastigote stage can upregulate Th2-associated cytokine production within macrophages and promote parasite survival and replication (107). Cytokines IL-4 and IL-13 can also drive an alternative activation of macrophages that promote Th2 effector responses and enhance parasite proliferation and survival (39, 108). Additionally, *L. major* persists in macrophages by expressing pathogenicity factors, such as lipophosphoglycan (LPG), that modify the phagosome into a parasitophorous vacuole (107). These modifications impair vacuole acidification and induce actin accumulation, creating a physical protective barrier around the vacuoles (107, 109). Moreover, GP63, a *Leishmania* protease, has been shown *in vitro* to affect intracellular signaling and transcriptional activities, leading to a decrease in TNF, IL-12, and NO secretion (110–112), thereby rendering the natural macrophage response insufficient to eliminate parasites. Contrary to these findings, however, *in vivo* GP63 has been shown to increase TNF and IL-6 production, while enhancing neutrophil and inflammatory monocyte influx to promote infectious spread (107, 113). These studies indicate that GP63 may impose varying effects depending on the cell type, and that the tissue microenvironments further dictate function to promote survival. More recently, the *L. major* Seidman stain, which causes non-healing lesions in C57BL/6 mice (114), was shown to preferentially infect mannose-receptor high M2-like dermal macrophages, which were locally maintained by IL-4 and IL-10 and were permissive to parasite growth (115). Remarkably, eosinophils were implicated in maintaining the M2-like phenotype of the tissue-resident macrophages (TRMs) through the production of IL-4 (116). In turn, IL-4 and IL-10-stimulated TRMs released CCL24, further recruiting eosinophils and reinforcing the amplification loop. The rapid recruitment of eosinophils and their close interaction with TRMs at both steady state and during infection was elegantly demonstrated by intravital microscopy using *eoCre il4/13f/f* mice, where IL-4 and IL-13 deficiency was selectively crossed under the endogenous eosinophil peroxidase promoter. As IL-10 was also required for CCL24 secretion by TRMs, it would be interesting to determine the source of this immunomodulatory cytokine, such as regulatory T cells.

## Th1 CD4^+^ T cells Are Indispensable for Leishmania Parasite Control

Th1 cells are responsible for the classical activation of macrophages, being the main source of IFN*γ* aside from Natural Killer cells (108). Th1 cells help infected macrophages clear *L. major* through the release of both IFN*γ* and TNF, which promote ROS and nitric oxide (NO) production to aid in killing intracellular parasites (117). Activated T cells also upregulate CD40L, which bind CD40 on macrophages and act as a secondary activation signal (118). To counteract T cell recognition, *L. major* utilizes several mechanisms that interfere with the antigen presentation machinery within infected macrophages (119–122). GP63 can cleave the CD4 molecule on T cells and suppress MHC-I presentation, physically disrupting CD4^+^ and CD8^+^ T cell activation, respectively (123). *Leishmania* antigens are sequestered from the MHC-II pathway (124) through fusion of the parasitophorous vacuole with the endocytic organelles to limit access to the antigen presentation machinery. This mode of immune evasion seems to be particularly important during the later stages of infection (120, 121). Similarly, infected macrophages activated the *Leishmania* homologue of receptors for activated C kinase (LACK)-specific CD4^+^ T cells at 6 hours post-infection, whereas T cell activation was drastically reduced at 24 hours and completely abolished at 48 hours post-infection (121), suggesting active suppression of MHC class II pathway. LACK genes are essential for parasite viability and share homologies with mammalian RACK (Receptors for Activated C Kinase) (125). They belong to the WD repeat protein family, playing a role in signal transduction, RNA processing, and cell cycle control and are essential for parasite survival (125). Subsequent reduction in TCR signaling and impaired reorientation of microtubule-organizing center (MTOC) towards the site of contact (120) helps explain poor T cell responses, but reduction in LACK expression at later timepoints cannot be ruled out, as macrophages infected with amastigotes were unable to stimulate LACK-specific T cells despite addition of stimulatory cytokines (121). *Leishmania* has also been shown to disrupt lipid rafts that are enriched in peptide/MHC-II complex, thereby decreasing TCR activation threshold (119, 120). But how do these data translate to the anti-*Leishmania* T cell responses *in vivo*? 2PTM studies visualized, for the first time, that while *Leishmania*-specific and non-specific CD4^+^ T cells both homed into infected skin of mice, *Leishmania*-specific T cells displayed reduced cell migration speeds and accumulated at the site of infection (126), based on their confined motility behaviors. Interestingly, only a subset of T cells formed stable contacts with infected cells (126), suggestive of either physical or immunological barriers that resulted in incomplete effector responses. This was not due to the background strain of mice used [Balb/c, producing a predominantly Th2 response to *Leishmania* infection (127)] because similar T cell behaviors were observed with Leishmania-specific T cells isolated from Th1-dominant C57Bl/6 mice. Studies by Muller et al. showed that paracrine expression of IFN*γ* by T cells can activate infected macrophages up to 80 microns away from the site of macrophage:T cell interactions (128). These studies argue that while *L. major* employs multiple mechanisms to suppress effector T cell responses, parasite control can be achieved with activation of as little as 10% of parasite-specific CD4^+^ T cells. Interestingly, Th1 and Th2 cell subsets are endowed with distinct motility programs that regulate their movement in inflamed skin (129). While Th1 cells are dependent on G protein-coupled receptor signaling that facilitate chemokine-driven migration, Th2 cells upregulate integrin α_V_β_3,_ allowing them to scan a larger tissue area independent of chemokine gradients (129). These studies indicate that Th1 cells are specifically programmed to sense changes in the environmental milieu during *Leishmania* infection to maximize effector responses. Despite this, small pools of persistently infected cells remain in the skin (discussed below), arguing that additional mechanisms are in place that prevent complete parasite clearance *in vivo*, even in the face of strong cellular immunity induction (121).

## Visualizing Immunity to Cutaneous Leishmaniasis in the Skin During Chronic Infection

### Long-Term Persistence of *Leishmania* Parasites *In Vivo*

BALB/c mice are known to develop progressive non-healing lesions, but even the resistant C57BL/6 strain takes weeks to heal and a small number of parasites continues to survive in healed skin (38). Persistent *Leishmania* parasites in the tissue post lesion-resolution appear to play a role in the maintenance of memory and protective immunity against reinfection (48, 100), but it is not clear whether long-term parasite survival is due to incomplete clearance by T cells or existence of sanctuary cells that are more hospitable for parasites. Studies from the Beverley lab described two distinct populations of the persistently-infected macrophages in the skin. One subset contained parasites that remain quiescent, whereas parasites in another subset continued to replicate in a manner similar to those during the acute stage of the infection (100). The fact that the number of parasites remain unchanged during the chronic stages of infection strongly suggests that a state of equilibrium between proliferation and immunity has been established. This is consistent with the fact that a complete clearance of parasites results in a total loss of immunity (50, 130). These studies also showed that persistent parasites were found within iNOS^+^ host cells, which were previously thought to completely clear parasites through NO production (100). This observation argues against the presence of “sanctuary sites” that can accommodate long-term *L. major* infections in the skin. A possible explanation is that either NO preferentially kills metabolically active parasites, or that persistent parasites are resistant to iNOS-mediated killing (100). Alternatively, continual low influx of susceptible inflammatory monocyte may contribute to host cell reservoir upon infection and maturation (45). These studies highlight possible cell intrinsic and extrinsic mechanisms that promote persistent infection that have the potential to re-established active disease, and questions remain why strong cell-mediated immunity are not able to completely remove infection.

Upon resolution of primary *Leishmania* infection, patients exhibit a long-lasting, CD4^+^ T cell-dependent concomitant immunity to reinfection, but 10^2^ - 10^4^ parasites remain detectable at the site of primary infection and in the draining lymph nodes (48, 53, 100, 131). These parasites seem vital in maintaining memory CD4^+^ T cells that can rapidly respond to subsequent *Leishmania* challenge. However, unlike generation of long-lived CD8^+^ T cells during viral infection, maintenance of memory CD4^+^ T cells against intracellular parasites remains poorly understood. Effector memory T cells (T_EM_) are generated during primary infection, yet it is currently not clear whether these cells also require a small pool of parasites for their long-term maintenance (52, 132). Central memory T cells (T_CM_) home to secondary lymphoid organs, such as the draining lymph node, where they proliferate and differentiate into effector T cells and migrate back to the lesion sites to mediate effector activities (52). T_CM_ are thought to serve as a long-lived source of *Leishmania*-specific effector T cells upon re-challenge and have been shown to survive in the absence of parasites (52), although this has not been observed in other studies (51, 130). Adoptive transfer of memory CD4^+^ T cell populations into naïve mice leads to their recruitment and enhanced parasite control upon *Leishmania* challenge (52, 53), but do not provide the same level of protection as those in immune animals. One possible explanation is the establishment of CD4^+^ T_RM_ in skin that facilitate recruitment of circulating memory T cells (133) and inflammatory monocytes (134) to aid in parasite clearance, the latter through ROS and iNOS production. Rapid influx of short-lived CD4^+^Ly6C^+^Tbet^hi^ T cells that are not derived from reactivated memory T cell pools have also been described to facilitate parasite clearance upon secondary challenge (53), highlighting the contributions from multiple immune subsets towards anti-*Leishmanial* immunity. In contrast, regulatory T cells are present in healed lesions, and can suppress effector CD4^+^ T cell function through IL-10 dependent and independent mechanisms (130). Sterilizing immunity can be achieved upon *in vivo* depletion of regulatory T cells (130), strongly arguing that Tregs are important in establishing a tolerogenic environment that promote long-term parasite survival. Consistent with this, healed mice challenged with heat-killed *Leishmania* parasites led to the rapid expansion of IL-10-producing regulatory T cells resulting in disease (lesion) reactivation (62). These data argue that an equilibrium is established between regulatory and effector T cells that help maintain a pool of persistently infected macrophages, and that disruption of this balance can re-establish infection (63, 135). What remains to be established is the dynamic interplay between regulatory T cells and effector CD4^+^ T cells within healed skin that contribute to parasite persistence *in vivo*, and whether Tregs need to see *Leishmania* antigen to establish a tolerogenic environment (136). 2PTM studies of healed skin will undoubtedly provide unprecedented insights into physical and cellular barriers that prevent complete parasite clearance and will provide insights into the barriers in place to achieve sterilizing, long-lasting immunity.

## Concluding Remarks

Parasites have evolved sophisticated mechanisms to evade detection from the immune system and to survive under harsh conditions. Some of the challenges against developing a vaccine against vector-borne infections, such as *Leishmania* spp., include the complexity in its route of infection, digenetic life cycle and their ability to counteract host immunity to establish a chronic infection. Intravital microscopy of infected mice has allowed for comparative analyses of dynamic host responses at various stages of disease in multiple organ sites. Yet, there is conflicting data on the contribution of immune subsets to protective immunity, as this seems to vary depending on the route of infection and the species used for infection studies. Future studies must compare and contrast immune responses to different parasite species, with an emphasis on why parasites can establish a persistent infection despite generation of a strong Th1 response. Intravital studies will be instrumental in revealing immune players, such as eosinophils or regulatory T cells, that are responsible for generating and maintaining a permissive environment that promote long-term parasite survival. Visualizing complex behaviors such as neutrophil extravasation from blood and their swarming at the site of bite injury (17), *L. major* transmission between cells through efferocytosis (104), and dynamic capture of *L. major* promastigotes by migratory dermal DCs in the skin (90) can only be accomplished by IVM directly in living tissues, and helps illuminate dynamic host:parasite interactions *in vivo* that cannot be captured by conventional, static approaches. Integrating intravital microscopy with other novel approaches, such as spatial transcriptomics analysis, will help reveal how tissue heterogeneity and gene expression impacts cellular behaviors to describe mechanisms in place to ensure parasite survival. These and other complementary approaches may translate into the development of new therapeutics against parasitic infections.

## Author Contributions

Conceptualization RZ and TM. Writing – original draft preparation RZ and TM. Writing – review and editing RZ, TM, and JU. Figures RZ. Supervision TM. Funding acquisition JU and TM. All authors contributed to the article and approved the submitted version.

## Funding

This work was supported by the CIHR-GSK Partnered Program (TM) and a studentship from Research Manitoba and University of Manitoba Student Fellowship (RZ). JU is supported by a CIHR Project grant MOP 114932.

## Conflict of Interest

The authors declare that the research was conducted in the absence of any commercial or financial relationships that could be construed as a potential conflict of interest.
